# Effect of thermomechanical loading on fracture resistance and failure mode of new pressable zirconia-reinforced lithium disilicate onlay restoration

**DOI:** 10.34172/joddd.40843

**Published:** 2024-03-29

**Authors:** Walid A. Abdelhady, Mohamed F. Metwally, Khaled M. Haggag

**Affiliations:** Crown and Bridge Department, Faculty of Dental Medicine, Al Azhar University, Cairo, Egypt

**Keywords:** Fracture resistance, Lithium disilicate, Onlay restorations, Thermomechanical loading, Zirconia-reinforced lithium disilicate

## Abstract

**Background.:**

Insufficient information exists regarding the fracture resistance and failure pattern of newly developed zirconia-reinforced lithium disilicate (ZL, Vita Ambria) onlays. This in vitro study compared the fracture resistance of two types of onlays: monolithic lithium disilicate (LD) and monolithic ZL.

**Methods.:**

Forty-eight ceramic onlay restorations were fabricated on epoxy dies using a maxillary first premolar model. The samples were divided into two main groups: LD and ZL. Half of each group was subjected to thermomechanical fatigue loading (TML) using a chewing simulator. All the samples were cemented with self-adhesive resin cement. Subsequently, they were loaded until failure in a universal testing machine, and the fracture patterns and resistance were recorded.

**Results.:**

Before TML, ZL demonstrated the highest statistically significant mean fracture resistance (499.76±34.14N) compared to LD (470.40±27.38N). After TML, ZL showed the highest non-statistically significant mean fracture resistance (429.27±131.42N), while LD’s mean fracture resistance decreased (377.31±62.18N).

**Conclusion.:**

Monolithic zirconia-reinforced onlays demonstrated higher fracture resistance and a more favorable failure mode compared to LD. However, the impact of thermomechanical aging resulted in reduced fracture resistance for both materials, with a notable preference observed for ZL.

## Introduction

 Contemporary dentistry emphasizes the concept of “minimally invasive procedures,” striving to preserve maximum tooth structure whenever possible. However, significant advancements in direct resin composites are still preferable for smaller defects. Moreover, their use in the posterior region is still accompanied by challenges, including high polymerization shrinkage, gap formation, occlusal wear, and color instability. As an alternative to direct partial restorations, indirect restorations have become more common due to their capacity to achieve superior control over the desired form and esthetics,^1^ particularly when dealing with larger defects in posterior teeth.

 In recent years, the invasiveness associated with indirect restorations has considerably decreased. This is supported by measurements of hard tissue removal using different preparation geometries. Meanwhile, full crowns in the anterior and posterior regions might require removing up to 70% of the clinical crown’s hard tissue.^2^ The tissue loss is significantly lower for partial crowns and occlusal onlays.^3^ As a result, these findings progressively influence treatment decisions favoring indirect restorations.^4^

 In the field of fixed prosthetics, a shift towards less invasive treatment concepts has become evident in recent years. Indirect partial coverage ceramic onlays have attracted attention as a more conservative choice in contrast to complete coverage crowns, resulting in substantial preservation of the remaining tooth structure.^1,5^ This is facilitated by advances in luting procedures, enabling the creation of onlays with reduced retention forms. Furthermore, research indicates that these onlays demonstrate enhanced mechanical properties, making them more fracture-resistant.^6^ Moreover, the surge in demand for ceramic onlay restorations can be attributed to the substantial advancements in ceramic materials used for dental restoration.^7^ Manufacturers have achieved enhanced mechanical and optical properties in ceramics by incorporating filler particles, such as leucite, zirconia particles, and lithium disilicate (LD) crystals, into the base glass composition.^8^

 IPS e.max Press by Ivoclar is a particle-filled glass ceramic with a high content of LD crystals, contributing to its enhanced mechanical properties. This type of glass ceramic was first introduced in the late 1990s as conventionally pressed LD copings, which are then layered with compatible porcelains.^9^

 Many manufacturers have developed various derivatives of LD to enhance its properties. One such derivative is zirconia-reinforced glass ceramic, which combines the favorable characteristics of glass and zirconia ceramic.^10^ Among these derivatives, lithium silicate (Li_2_O_3_Si)-reinforced ceramics enriched with 10% zirconia, such as Suprinity and Celtra Duo, have been introduced. These ceramics exhibit higher flexural strength and improved esthetic properties.^11,12^ A recently invented pressing glass ceramic with 8‒12% zirconia oxide, VITA AMBRIA^TM^ by Vita Zhanfabrik, has entered the market. This innovative material is indicated for constructing crowns, onlays, and veneers.^13,14^

 Several investigators have observed that fracture is the most frequent reason for replacing dental prostheses. Therefore, assessing a dental material’s fracture resistance is crucial before employing it as a long-term permanent restoration in different clinical conditions.^15^ Several factors can influence the results of the fracture resistance test, including the material’s composition, mechanical characteristics, and the applied load on the restoration.^16-18^ The fracture resistance test can assist in identifying the force that could break the tooth‒restoration complex, thereby suggesting optimal preparation designs and restorative materials with the greatest resistance to fracture.^19,20^

 In a literature review assessing the long-term clinical longevity of various restorative materials, ceramic onlays and inlays exhibited an annual failure rate ranging from 0% to 7.5%.^1^ However, Vita Ambria is a relatively new material with inadequate previous studies evaluating its long-term performance and fracture resistance. For this reason, the main objective of this study was to assess the fracture resistance of maxillary premolars restored with Vita Ambria and IPS e.max press onlay restorations. The null hypotheses of this study were as follows: [1] No differences will be found in fracture resistance between the two tested materials before and after the thermomechanical fatigue loading (TML). [2] TML will not affect the fracture resistance of each material.

## Methods

**Figure 1:**
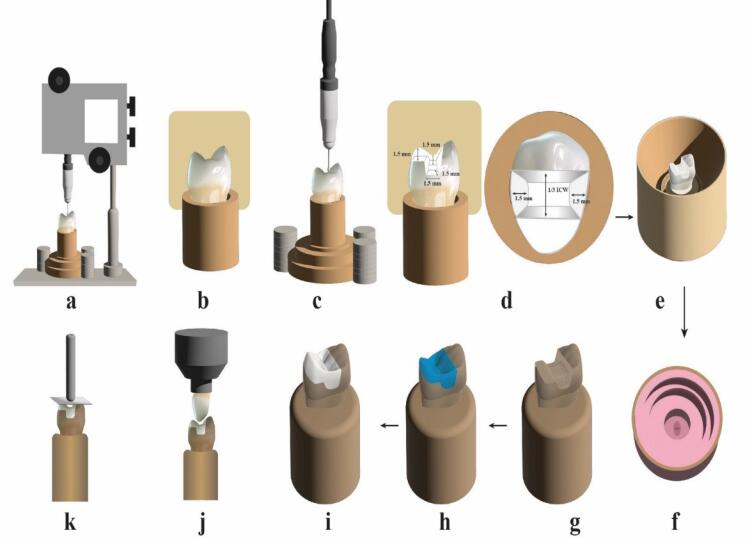
Schematic flowchart illustrating the study procedures

 The sample size was calculated from a power test based on the results of Al-Akhali et al.^21^ with β = 0.80 and α = 0.05. A sample size of n = 24 for each main group was determined. Forty-eight ceramic onlay restorations were fabricated; they were divided into two equal main groups (n = 24), each according to the type of ceramic material used: group LD, lithium disilicate (IPS e.max Press), and group ZL, zirconia-reinforced lithium disilicate (Vita Ambria). Then, half of each main group was subjected to thermomechanical aging, and the other subgroup was not aged (n = 12). A schematic flow chart of the experimental procedure is shown in Figure 1. The study overview included: (a) Fixing the typodont tooth with a parallelometer in the custom holder. (b) Carrying out the putty index to control the preparation. (c) Performing tooth preparation with the aid of a milling surveyor. (d) The preparation dimensions. (e) Seating the metal ring around the master die and pouring the duplicating silicon material. (f) Preparing the silicon mold for pouring the epoxy resin. (g) The epoxy resin dies after removal from the silicon mold. (h) Designing and milling the wax pattern using a computer-aided design and computer-aided manufacturing (CAD/CAM) system. (i) Fabricating (pressing) the onlays from each material. (j) Performing thermomechanical loading test for the aged groups. (k) Loading the samples to failure and recording the fracture resistance values.

###  Master die fabrication and onlay preparations

 To conduct the present in vitro study, a maxillary first premolar typodont tooth was selected and embedded, with the aid of a parallelometer (Paraflex, Bego, Bremer, Germany), using auto-polymerizing polymethylmethacrylate inside a special cupper holder, which was fabricated to hold and fix the tooth during preparation with their long axes oriented perpendicular to the surface of the block up to 3 mm below their cementoenamel junction (CEJ) to simulate alveolar bone level (Figure 1a).

 Regarding standardization, all the samples from both tested groups were identical as they were duplicated from the master die using shrinkage-free silicone duplication material, which was used to create 48 silicone molds. These molds were then poured with epoxy resin material. Therefore, the onlay preparation was standardized for all the samples. The onlay preparation for the master die was meticulously controlled during the reduction process to achieve specific dimensions. This control was exercised using the milling surveyor (Paraskop® M, Bego, Bremer, Germany) (Figure 1c) and the silicone putty index (Zeta plus; C-Silicone, Zhermack) (Figure 1b) to attain the following dimensional parameters: The pulpal floor depths were prepared to a depth of 1.5 mm from the occlusal cavosurface margin of preparations. The axial wall was prepared with a 10° divergence. A reduction of 1.5 mm on the functional cusp was established. The widths of the gingival floor preparations were set at 1.5 mm with a depth of 1.5 mm. The width of the occlusal isthmus was determined to be 1/3 the width of the intercuspal distance. Internal line angles were rounded to smooth the preparations (Figure 1d).

###  Duplication of the master die

 A customized metal ring was positioned over the master die, and 48 silicon molds were taken for the master die with a shrinkage-free silicon duplicating material (Replisil; Zubler, USA, Dallas, TX) and mixed according to the manufacturer’s recommendations (Figure 1f). The silicone mold was poured with epoxy resin (Kemapoxy 150; CMB International, Egypt), poured into it under vibration, and left to polymerize for 24 hours. The epoxy resin die was then removed, and 48 epoxy dies were made (Figure 1g).

###  Construction of onlay restorations

 Forty-eight biogeneric wax patterns were made with an Amann Girrbach (Amann Girrbach Vorarlberg, Austria) CAD/CAM system.^22^ All fabrication steps followed the manufacturer’s recommendations. The master die was sprayed with the powder Shera scan spray (Shera Werkstoff-Technologie, Germany) to remove optical highlights from the surface of the die and enhance the precision of the optical impressions acquired by creating a uniformly reflective surface. An optical impression was taken with a Ceramil Map 400 scanner (Amann Girrbach, Vorarlberg, Austria). After evaluating the clarity of the scan, the data were stored using the computer software provided by the manufacturers. On the computer screen, a 3D model was created by Ceramil Mind software integrated with the in-lab Amann Girrbach CAD/CAM system. The design was performed through different steps, starting with (margin detection) margins that were delineated in automatic mode and corrected manually when necessary, followed by creating the virtual design according to the following parameters: minimum radial thickness of 1.5 mm; minimum occlusal thickness of 1.5 mm; a 50-μm cement gap, an adhesive gap of 100 μm, and a margin thickness of 120 μm.

 After the restorations had been designed, the milling preview window was activated to start the milling process. The Ceramil motion of the two milling machines was then activated, the Ceramil wax blank was fixed in the milling machine’s spindle, and the door was closed. Then, the milling icon was clicked to start the milling process; the wax patterns were separated from the disc at the end of milling and evaluated on the master die. Wax patterns were also inspected on their corresponding epoxy die for proper seating before investing (Figure 1h). The lost wax technique was used in this study to fabricate onlay restorations by pressure injection of ceramic ingots in the EP500 furnace (Ivoclar Vivadent) following the manufacturer’s recommendations for each material. Twenty-four monolithic ceramic onlays (n = 24) were fabricated from each material: ZL and LD.

###  Cementation of the restorations

 The intaglio surface of each restoration was etched with 9.5% hydrofluoric acid (Porcelain etchant; Bisco, Inc. Schaumburg, USA) for 20 seconds, thoroughly rinsed with water, and air-dried. The etched surface was coated with a silane (Porcelain primer; Bisco, Inc. Schaumburg, USA), applied with a brush, and air-thinned after one minute. Self-adhesive resin cement (TheraCem; Bisco, Inc. Schaumburg, USA) was used to cement the onlays. The activation, mixing, placement, and polymerization were carried out according to the manufacturer’s recommendations. Each restoration was seated on its corresponding epoxy resin die and fixed to a specially designed cementation device for load application (49 N) during the cementation procedure (Figure 1i and Figure 1j). After cementation, samples were stored in distilled water at 37 °C.

###  Testing procedures 

 Half of the samples in each main group (n = 12) were subjected to thermomechanical cyclic loading via cyclic load multimodal ROBOTA chewing simulator (ROBOTA Chewing Simulator, Model ACH-09075DC-T, Germany) integrated with a thermocycling protocol operated on servomotor (Figure 1j). Each sample underwent 120,000 preloaded cycles accompanied by 10000 thermal cycles (5‒55 ºC), a dwell time of 60 seconds, and a load of 98 N.^23^ Force was applied to a palatal cusp of the tooth structure centrally on the occlusal surface of the onlays attached to the upper movable compartment of the material testing machine.

 The samples were mounted on a computer-controlled materials testing machine (Instron universal testing machine, Model 3345, England)with a load cell of 5 KN, and data were recorded using computer software(Bluehill 3 software version 3.3.). Samples were secured to the lower fixed compartment of the testing machine by tightening screws. The fracture test was performed by compressive mode of load applied occlusally on the central fossa using a metallic rod with a round tip (3.6 mm in diameter) attached to the upper movable compartment of the testing machine, moving at a crosshead speed of 1 mm/min.^19,24^ A 1-mm-thick tin foil sheet was placed between the sample and the metallic rod to achieve homogenous stress distribution and minimization of the transmission of local force evenly^19^ (Figure 1k). The load at failure manifested by an audible crack and confirmed by a sharp drop at the load-deflection curve recorded using computer software. The load required to fracture was recorded in Newton (N).

###  Failure mode assessment

 After the fracture resistance test, the fractured samples were examined to determine failure patterns using a USB digital microscope (U500x Digital Microscope, Guangdong, China). The fracture mode for each tooth was classified as follows^25^:

- Mode I: Extensive crack formation within the ceramic
- Mode II: Cohesive fracture within the ceramic
- Mode III: Fracture within the ceramic and tooth structures above the CEJ
- Mode IV: Longitudinal ceramic and tooth fracture below the CEJ.

Statistical analyses were performed using a commercially available software program (SPSS Chicago, IL, USA, version 20). Numerical data were summarized using means, standard deviations, and confidence intervals. Data were explored regarding normal distribution using Kolmogorov-Smirnov and Shapiro-Wilk tests. Comparisons between groups concerning normally distributed numeric variables were compared by independent *t *test. Comparison before and after thermomechanical loading was performed by paired *t* test. Two-way ANOVA was used to study the effect of the group and the loading variables. Qualitative data were described as frequency (number) and percentage. Data were compared using the chi-squared test. The level of significance was set at *P* ≤ 0.05. All the tests were two-tailed.

## Results

**Figure 2:**
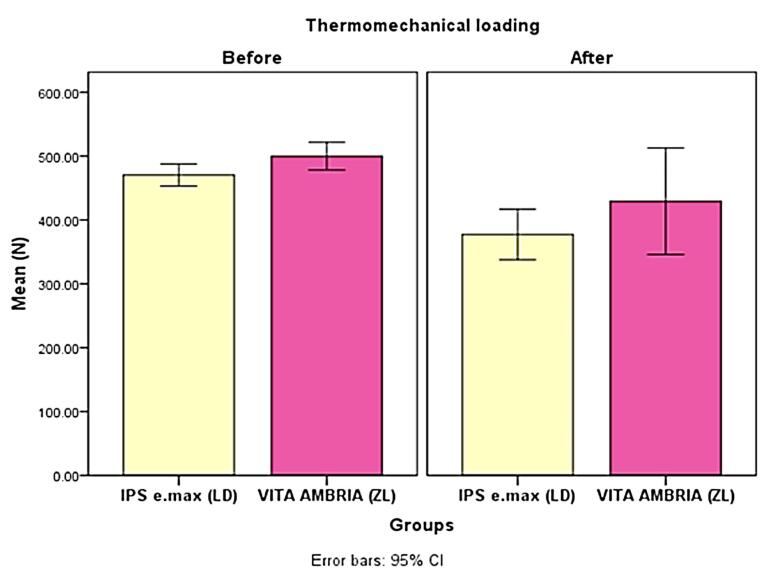
Bar chart illustrating the mean fracture load (N) between the groups. LD, lithium disilicate; ZL, zirconia-reinforced lithium disilicate

**Figure 3:**
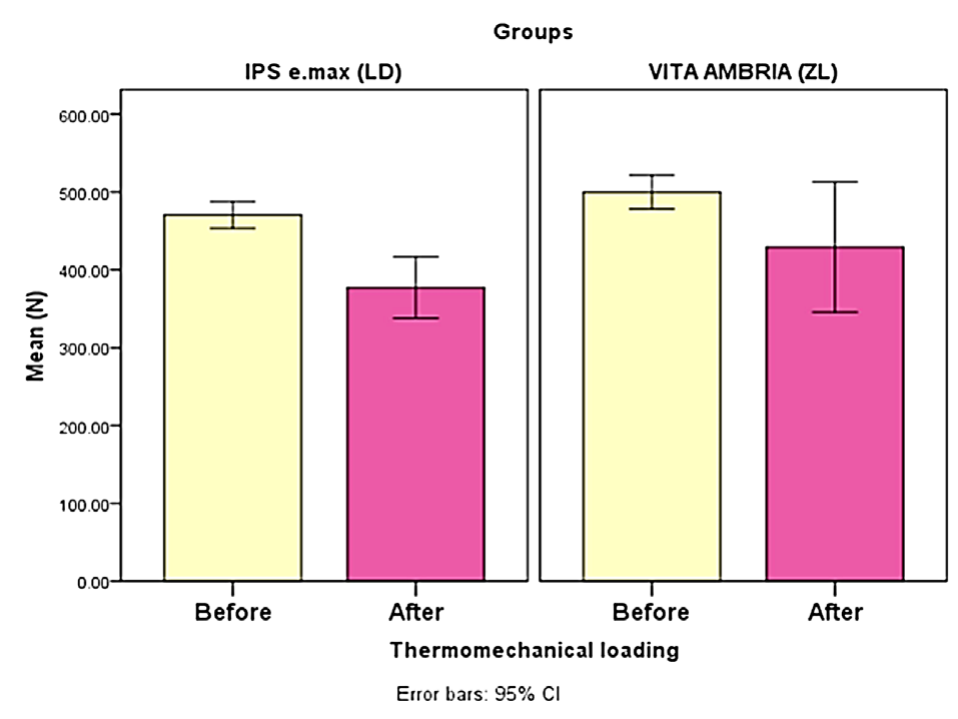
Bar chart illustrating the mean fracture load (N) effect of thermomechanical fatigue loading. LD, lithium disilicate; ZL, zirconia-reinforced lithium disilicate

 The results of the fracture resistance of the groups are listed inTables 1 and 2 and Figures 2 and 3. When comparing the groups without TML, the ZL showed significantly higher fracture resistance than LD (*P* = 0.03). After TML, the ZL showed a higher non-statistically significant difference than LD (*P* = 0.234) (Table 1 and Figure 2).

**Table 1:** Descriptive fracture load (N) statistics and comparison between groups (independent t-test)

**Group/TM loading**	**Mean**	**SD**	**Difference between groups**				**t**	***P*** ** value**
			**Mean**	**Std. Error**	**95% CI**			
					**Lower**	**Upper**		
LD Without TML	470.40	27.38	-29.36	12.63	-55.56	-3.16	2.32	0.03*
ZL Without TML	499.76	34.14						
LD After TML	377.31	62.18	-51.96	41.97	-141.1	37.16	1.23	0.234 ns
ZL After TML	429.27	131.42						

LD, lithium disilicate; ZL, zirconia-reinforced lithium disilicate; SD, standard deviation; ns, non-significant,CI, confidence interval; TML, thermomechanical fatigue loading. *Significant, significance level *P* ≤ 0.05.

**Figure 4:**
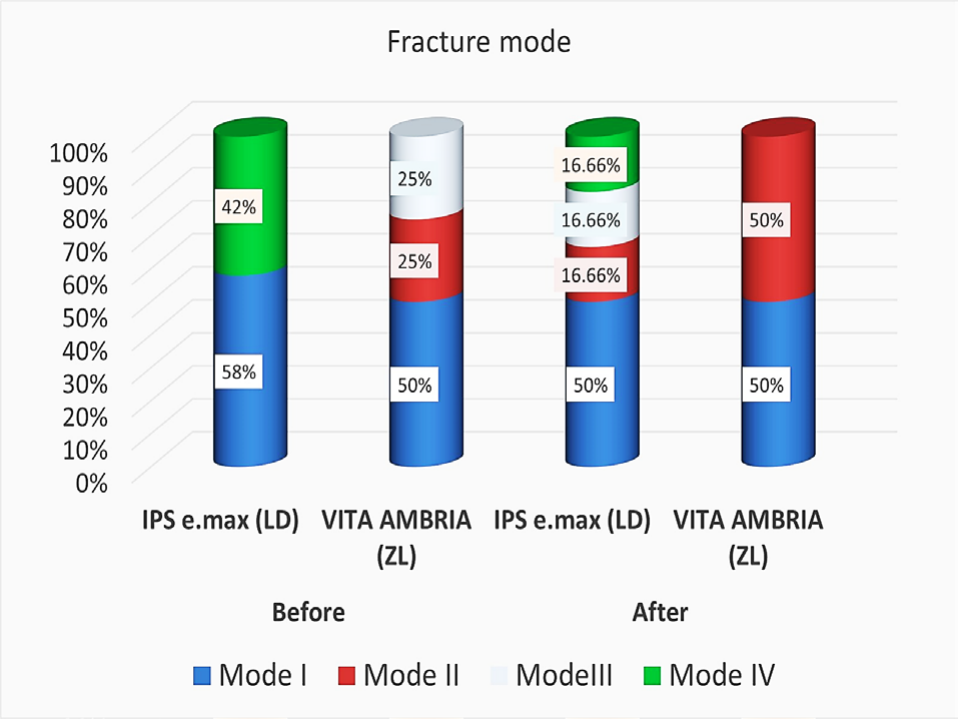
Bar chart illustrating the distribution of failure mode before and after thermomechanical loading in both groups.

 Regarding the TML effect, although all aged specimens survived the TML without fracture, ceramic chipping, and cracks, TML decreased the fracture resistance significantly for LD (*P* = 0.00), and non-significantly for ZL (*P* = 0.086) (Table 2 and Figure 3). In the current study, the failure mode after the fracture test was evaluated and represented descriptively in (Figure 4). It was remarkable that the most common failure modes for non-age groups were modes I and IV for LD and modes I, II, and III for ZL groups. After TML, the failure mode showed different modes with LD, with modes I and II for the ZL group.

**Table 2:** Descriptive fracture load (N) statistics and comparison within a group without and after thermos-mechanical loading (paired t-test)

**Group/TM L**	**Mean**	**SD**	**Mean**	**Std. Error**	**95% CI **		**t**	***P*** ** value**
					**Lower**	**Upper**		
LD without TML	470.40	27.38	93.09	19.61	52.41	133.77	4.75	0.00*
LD after TML	377.31	62.18						
ZL without TML	499.76	34.14	70.49	39.20	-10.80	151.78	1.8	0.086 ns
ZL after TML	429.27	131.42						

LD, lithium disilicate; ZL, zirconia-reinforced lithium disilicate; SD, standard deviation; ns, non-significant,CI, confidence interval; TML, thermomechanical fatigue loading. *Significant, significance level *P* ≤ 0.05.

## Discussion

 Fracture is the primary cause of failure of ceramic onlay restorations.^26,27^ This study investigated the fracture resistance of onlay restorations fabricated from two heat-pressed ceramic systems. Based on the results of this study, since all the samples survived the TML (thermomechanical loading), and the mean fracture resistance values were significantly affected by the TML test, the first part of the first null hypothesis, which stated that no differences would be found in fracture resistance between the two tested materials before and after TML, was partially accepted, as the material type had a significant effect before TML. The second null hypothesis, which stated that the fracture resistance of each material itself would not be affected, was rejected.

 Many variables can affect the fatigue and fracture behavior of ceramic restorations, including the restoration material, die material, preparation geometry, thickness of the restoration, and cementation procedures.^19^ It is evident that natural tooth material appears to significantly influence stresses in loaded restorations, so it is crucial to consider this behavior when experimentally simulating the in vivo situation. An epoxy resin die with a lower elasticity modulus was used as a supporting structure for the fracture resistance test to simulate clinical conditions, allowing standardization of all samples and avoiding difficulty in reproducibility and comparability with natural teeth.^18^ In this study, to standardize the preparation design for all the samples, the master die was duplicated to identical epoxy resin dies using duplicating silicon material. To control the ceramic thickness of the restorations, one virtual restoration design was created using a CAD/CAM system and milled from a wax blank for all the samples to be pressed by ceramic ingots later.^22^

 This new ZrO_2_-reinforced lithium silicate pressable glass–ceramic system (Vita Ambria) introduced by Vita Zahnfabrik (Bad Säckingen, Germany) is enriched with approximately 8‒12 wt% ZrO_2_ particles. This newly developed generation of glass–ceramic combines the positive material characteristics of ZrO_2_ (high strength) and glass–ceramic (appealing aesthetics).^14^

 Thermocycling and dynamic loading are crucial in evaluating novel dental materials under accelerated conditions, simulating actual intraoral activities. The aged samples were subjected to cyclic loading for 120 000 cycles and 10 000 thermal cycles, where temperature changes from 5 to 55 in a thermocycling machine to mimic the temperature changes that may occur in the oral cavity due to hot and cold extremes. This simulation aimed to replicate one year of masticatory conditions in the posterior area of the oral cavity.^23^

 A compressive load was applied with a metallic rod with a round tip (3.6 mm in diameter), and a thin layer of tin foil was placed between the sample and the metal rod to allow better stress distribution inside the sample.^16^

 The fracture resistance for both tested groups showed fracture mean values exceeding the required physiological forces (300 N) even after the TML. Before TML, the ZL material exhibited significantly higher mean fracture resistance (*P* = 0.03) compared to monolithic LD. After TML, the ZL material exhibited a higher mean fracture resistance value than monolithic LD, but the difference was not statistically significant (*P* = 0.234). For these reasons, the first null hypothesis was partially accepted. This observation can be attributed to ZL’s inherent higher mechanical properties, as the added zirconia oxide (ZrO_2_, 8‒12%) works as a nucleating agent and increases the crystal gross. Huang et al^28^ studied the effect of different concentrations of ZrO_2_ added to LD glass ceramic and considered ZrO_2_ as a classic nucleating agent, and concluded that with low zirconia content (5−10 wt%), it acted as a nucleating agent, resulting in increased crystallinity and homogeneity of the material.

 Mavriqi et al^12^ tested the mechanical and microstructure properties after the crystallization of two zirconia-reinforced lithium silicate glass ceramics (Vita Suprinity and Celtra Duo) compared to IPS e.max CAD. Zirconia-reinforced LD glass ceramic showed more homogeneous lithium monociliate, aluminum silicate, and a glassy matrix enriched with tetragonal zirconia more evident in the post-crystallization state. LD is featured by needle-shaped crystals, interlocked, and embedded in the glassy matrix.

 Regarding the aging effect of TML on the tested materials, all the samples survived following TML. The LD group exhibited a statistically significant decrease in mean fracture resistance (*P =*0.000) compared to the non-aged LD group. In contrast, the ZL group showed a non-statistically significant decrease in mean fracture resistance (*P* = 0.086) compared with the non-aged ZL group. Consequently, the second null hypothesis, which postulated that TML would not affect the fracture resistance of each material, was partially rejected. This observation can also be attributed to the strong bond between the zirconia oxide and the resin cement containing 10-methacryloyloxydecyl dihydrogen phosphate (10-MDP). The functional monomer 10-MDP has been shown to form stable chemical bonds with zirconium atoms, which might eliminate the negative effects of aging and enhance the adhesive durability of resin and zirconia particles. The MDP monomer’s hydroxyl group (OH) can form a stable chemical bond with the hydroxyl group of zirconia and resist hydrolysis degradation. Furthermore, it can be assumed that the decyl group in MDP prevents water penetration at the interface between the dihydrogen phosphate and zirconia.^29^

 Moreover, due to crack deflection, the glass–ceramic might gain additional strength through ZrO_2_ transformation toughening. When the ZrO_2_ grain undergoes a phase transition, its volume expansion generates compressive stress on the crack or surrounding microcracks. This stress absorption by the main crack enhances the fracture resistance of the glass–ceramic. Also, the results are consistent with Asaka et al,^11^ who reported a notable increase in elastic modulus along with elevated fracture toughness and flexural strength compared to ZrO_2_-free LD glass–ceramic (IPS e.max CAD). These enhancements were attributed to the incorporation of zirconia filler into the glass matrix, which reinforced the material while avoiding clouding caused by dissolved zirconia particles, resulting in heightened fracture toughness.^11^

 Regarding the failure modes, ZL exhibited a more repairable failure mode than LD. For non-aged samples, ZL showed a distribution of 50% for mode I and equal 25% for modes I and III. After undergoing TML, ZL displayed an equal distribution of 50% for modes I and II. On the other hand, LD exhibited a distribution of 58% for mode I and 42% for mode IV (non-restorable) for non-aged samples. After TML, LD displayed all failure modes. These results may be explained as the ZL tends to have higher flexural strength and fracture toughness than LD, indicating that ZL is less likely to fail in a brittle manner and is better at resisting crack propagation, so that it might exhibit a more favorable failure mode. Standardization protocol was considered in the study, but limitations were unavoidable. Although in vitro simulations are useful for comparing materials in different situations, they cannot perfectly imitate real-life clinical conditions as in vivo studies. Moreover, restorations were tested on epoxy resin dies rather than natural teeth, potentially yielding different outcomes. For this reason, future studies should be supported by long-term follow-up of different clinical cases. Within the limitations of this study, the following conclusions can be drawn:

1. Both tested ceramic materials, LD (IPS e.max press) and ZL ceramic (Vita Ambria), exhibited fracture resistance values within the clinically accepted range.
2. Zirconia-reinforced glass ceramic showed better fracture resistance than LD glass-ceramic.
3. Zirconia-reinforced glass ceramic demonstrated a more favorable failure mode than LD glass‒ceramic.
4. TML negatively affected the fracture resistance of the tested materials, favoring ZL ceramic.

## Competing Interests

 No competing interests.

## Ethical Approval

 This study was approved by the Research Ethics Committee Faculty of Dental Medicine, Al-Azhar University, under the protocol number EC. Ref No: 679/272.

## Funding

 No funding.
